# The Role of FveAFB5 in Auxin-Mediated Responses and Growth in Strawberries

**DOI:** 10.3390/plants13081142

**Published:** 2024-04-19

**Authors:** Xuhui Wang, Shuo Feng, Jiangshan Luo, Shikui Song, Juncheng Lin, Yunhe Tian, Tongda Xu, Jun Ma

**Affiliations:** 1 College of Horticulture, Fujian Agriculture and Forestry University, Fuzhou 350002, China; 13123150031@163.com; 2 Plant Synthetic Biology Center, Haixia Institute of Science and Technology, Fujian Agriculture and Forestry University, Fuzhou 350002, China; 18241674166@163.com (S.F.); 13313918173@163.com (J.L.); shikui_song@163.com (S.S.); linjc2011@126.com (J.L.); yunhe_tian13@163.com (Y.T.)

**Keywords:** strawberry, auxin, FveAFB5, picloram, fruit development

## Abstract

Auxin is a crucial hormone that regulates various aspects of plant growth and development. It exerts its effects through multiple signaling pathways, including the TIR1/AFB-based transcriptional regulation in the nucleus. However, the specific role of auxin receptors in determining developmental features in the strawberry (*Fragaria vesca*) remains unclear. Our research has identified FveAFB5, a potential auxin receptor, as a key player in the development and auxin responses of woodland strawberry diploid variety Hawaii 4. FveAFB5 positively influences lateral root development, plant height, and fruit development, while negatively regulating shoot branching. Moreover, the mutation of *FveAFB5* confers strong resistance to the auxinic herbicide picloram, compared to dicamba and quinclorac. Transcriptome analysis suggests that FveAFB5 may initiate auxin and abscisic acid signaling to inhibit growth in response to picloram. Therefore, FveAFB5 likely acts as an auxin receptor involved in regulating multiple processes related to strawberry growth and development.

## 1. Introduction

The plant hormone auxin is a key player in various aspects of plant growth and development [1], such as embryogenesis [2,3], tropic growth [4], stem elongation [5], root elongation [6], the formation of leaves and flowers [7], and fruit development [8]. It exerts its effects through processes such as synthesis and metabolism, polar transport, and signal transduction pathways. The well-established canonical auxin signaling pathway involves the participation of the TRANSPORT INHIBITOR RESPONSE1 (TIR1)/AUXIN-SIGNALING F-BOX (AFB) proteins [9,10,11,12]. This pathway relies on crucial components, including the TIR1/AFB proteins, the Aux/IAA transcriptional repressors, and the ARF transcription factors. In this pathway, auxin promotes the protein interactions between Aux/IAA and SCF^TIR1/AFB^ ubiquitin E3-ligase complex, which leads to the degradation of Aux/IAA repressors, allowing the activation of ARFs and the subsequent transcriptional responses [9,10,13,14]. 

Auxin plays an essential role in strawberry growth and fruit development as well [8,15,16,17,18]. Fertilization-induced auxin biosynthesis is required for seed and fruit development [19]. Auxin biosynthesis genes, including *FveYUC4* and *FveYUC6,* are essential for leaf and flower morphogenesis in the strawberry [7]. Auxin-signaling genes *FaARF4* and *FveARF8* promote flowering and repress accessory fruit initiation, respectively, in strawberries [20,21]. However, it remains enigmatic how, as canonical auxin receptors, FveTIR1/AFB function during strawberry growth and fruit development. In *Arabidopsis*, there exist six members of the auxin receptor AtTIR1/AFB family, each demonstrating distinct biochemical properties and biological functions [22,23]. Notably, unlike other members of the family, AtAFB4 and AtAFB5 are indispensable to the response to the synthetic auxinic herbicide picloram [24,25]. Synthetic auxin herbicides, including phenoxy carboxylic acid (MCPA), benzoic acid (dicamba), pyridine carboxylic acid (picloram), and quinoline carboxylic acid (quinclorac), are extensively utilized in agriculture for their capacity to impede growth, disrupt plant physiology, and induce geotropism at elevated concentrations [26,27]. In rice, the *osafb4* mutant also exhibited strong resistance to the herbicide picloram [28]. In the woodland strawberry, three *FveTIR1/AFB* homologous genes are encoded in the genome: *FveTIR1*, *FveAFB2*, and *FveAFB5,* expressed in all fruit tissues; their functions are still unknown [18,29]. Thus, based on the relevant studies, it is probable that FveAFB5, which is the closest homolog of AtAFB5 and OsAFB4, contributes to strawberry development and resistance to auxinic herbicides.

In our study, we successfully generated *fveafb5* mutants and overexpression lines. Through our investigation, we have discovered that FveAFB5, which acts as a mediator of auxin signaling, plays a crucial role in regulating both plant growth and development. Notably, the *fveafb5* mutants exhibited robust resistance to synthetic auxinic herbicides, including picloram, dicamba, and quinclorac. The outcomes of our research promise to provide valuable insights into various aspects, such as plant growth and development, herbicide resistance, and effective weed management strategies specifically tailored for strawberries.

## 2. Results

### 2.1. FveAFB5 Is a Potential Auxin Receptor Homolog in Strawberries

The functional role of auxin is primarily mediated through a TIR1/AFB-dependent mechanism that regulates gene transcription [9,10,11,12]. However, how auxin receptor *FveTIR1/AFB* genes function during strawberry growth and development remains unknown. Combined with the published transcriptome data [18] and sequence similarity analysis, three members of the *TIR1/AFB* gene family in three distinct branches were identified, namely *FveTIR1*, *FveAFB2*, and *FveAFB5* (Figure S1A). Notably, all three proteins exhibit an amino-terminal, conserved F-box domain and four leucine-rich repeats (LRR) (Figure S1B). Through quantitative reverse transcription polymerase chain reaction (qRT-PCR), we discovered that *FveAFB5* displays high expression levels in flowers (Figure S2A), which slightly differs from the *FveAFB5* expression heatmap observed in *F. vesca* co-expression networks, which showed high *FveAFB5* expression levels in young leaf, flower, young fruit, and receptacle tissues (Figure S2B) [18,30,31]. The transcriptional difference may arise from the older tissues, such as mature leaves, and other factors, such as different varieties or growth conditions. Additionally, we also conducted subcellular localization studies using FveAFB5 fused with GFP and found that the protein is localized in the nucleus of *Nicotiana bentamiana* leaf epidermal cells (Figure S2C). 

The ability to bind to Aux/IAA proteins is an essential characteristic of auxin co-receptors [32]. To investigate whether FveAFB5 interacts with Aux/IAA proteins, we selected six FveIAA proteins that contain the conserved domain II according to a relevant study in tomatoes [33] and conducted bimolecular fluorescence complementation (BiFC) experiments on tobacco. The co-expression of nYFP-FveIAA4a/FveIAA11/FveIAA17/FveIAA27a and cYFP-FveAFB5 resulted in robust yellow fluorescent protein (YFP) signals within the nucleus (Figure S3), indicating an in vivo interaction between FveAFB5 and FveIAA4a, FveIAA11, FveIAA17, and FveIAA27a. These findings provide evidence for the physical interaction between FveAFB5 and specific FveIAA proteins, further supporting the role of FveAFB5 in modulating auxin signaling in strawberries. Collectively, these results indicated that FveAFB5, as a homolog of auxin receptors in strawberries, likely serves as a potential auxin receptor in strawberry growth and development.

### 2.2. FveAFB5 Is Required for Strawberry Growth and Development

**Figure 1:**
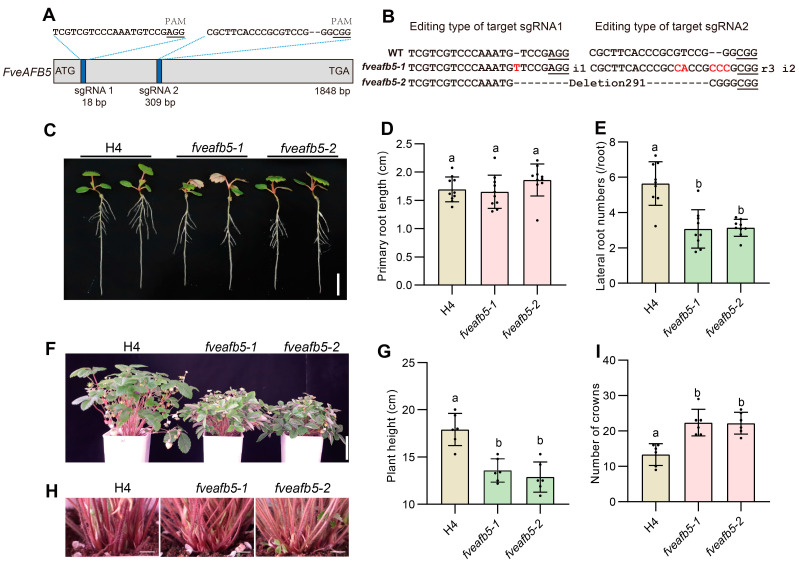
FveAFB5 is required for strawberry growth and development. (**A**,**B**) Mutation types of *fveafb5* mutants generated by CRISPR-Cas9 technology. (**A**) A schematic diagram showing the positions of CRISPR-Cas9 sgRNA; the blue boxes indicate the sgRNA1 and sgRNA2 sgRNA positions. The short horizontal lines indicate the PAM sites. (**B**) Mutation types of the *fveafb5* mutants. “i” represents insertion, “r” represents replace, and the red letters indicate the mutated bases. (**C**–**E**) Root phenotype (**C**) and quantification analysis of the primary root length (**D**) and lateral root density (**E**) in *fveafb5* mutants. (**F**,**G**) Plant height phenotype (**F**) and quantification analysis (**G**) in *fveafb5* mutants. (**H**,**I**) Shoot branching phenotype (**H**) and quantification analysis (**I**) in *fveafb5* mutants. Scale bars: 0.5 cm (**C**), 5 cm (**F**), and 1 cm (**H**), respectively. One-way ANOVA and different letters indicate significant differences at *p* < 0.0001, *n* = 9 (**D**,**E**), *n* = 5 (**G**), and *n* = 6 (**I**). The experiments were repeated three times.

To assess the functionality of the putative auxin receptor FveAFB5 in strawberries, we constructed CRISPR-Cas9 knockout and overexpression vectors, which were subsequently stably transformed into the woodland strawberry variety Hawaii 4. Through this approach, we successfully generated *FveAFB5* knockout mutants and overexpression lines for further investigation (Figure 1A,B and Figure S4A–C). It was observed that *fveafb5-1* and *fveafb5-2* represented two independent alleles with distinct mutations (Figure 1B). While both *fveafb5-1* and *fveafb5-2* mutants displayed normal primary root length, there was a significant decrease in lateral root density compared to the wild-type H4 (Figure 1C–E). Furthermore, the *fveafb5* mutants exhibited reduced plant height, increased branching, and smaller leaves in comparison to the wild type (Figure 1F–I). Conversely, the overexpression of *FveAFB5* resulted in a significant increase in lateral root density, indicating the opposite effect to that observed in the mutants (Figure S4D,E). Overall, these findings suggest that FveAFB5 plays a positive regulatory role in lateral root development and plant height while acting as a negative regulator of shoot branching in strawberries. Importantly, all these processes are closely associated with auxin signaling.

### 2.3. FveAFB5 Is Required for Strawberry Receptacle Development

**Figure 2:**
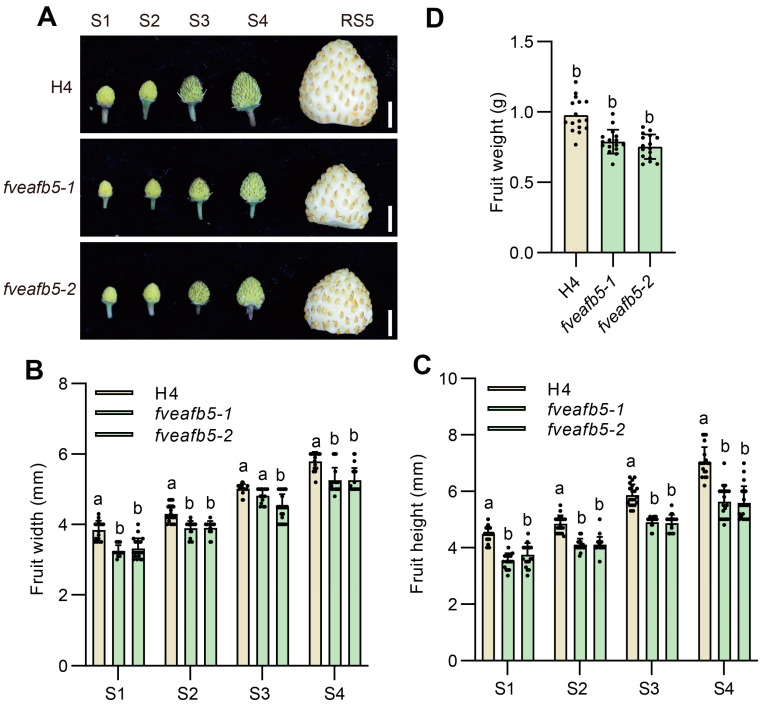
FveAFB5 is required for strawberry receptacle development. (**A**) Fruit morphology at different fruit developmental stages of H4 and *fveafb5* mutants. Scale bar: 0.5 cm. (**B**,**C**) Quantification of fruit width (**B**) and height (**C**) at S1 to S4 stages of H4 and *fveafb5* mutants. (**D**) Quantification of fruit weight at RS5 stages of H4 and *fveafb5* mutants. One-way ANOVA (**D**); two-way ANOVA (**B**,**C**); different letters represent significant differences at *p* < 0.0001 (*n* = 10–20). The experiments were repeated three times.

Auxin has been shown to play a pivotal role in controlling fruit development in strawberries [16,19,21]. To investigate the impact of FveAFB5 on fruit development, we examined the S1 (unpollinated) to S4 fruit development stages in the *fveafb5* mutants. Our findings revealed that the fruit height and width of the *fveafb5* mutants were significantly lower compared to those of the wild type (Figure 2A–C). Additionally, the fruit weight at the RS5 stage (fully ripe stage) of the *fveafb5* mutants was noticeably lighter (Figure 2A,D). Taken together, these results highlight the potential role of FveAFB5 in strawberry receptacle development. This intriguing outcome prompted us to further explore the downstream genes that are regulated by FveAFB5 during the early stages of strawberry fruit development.

**Figure 3:**
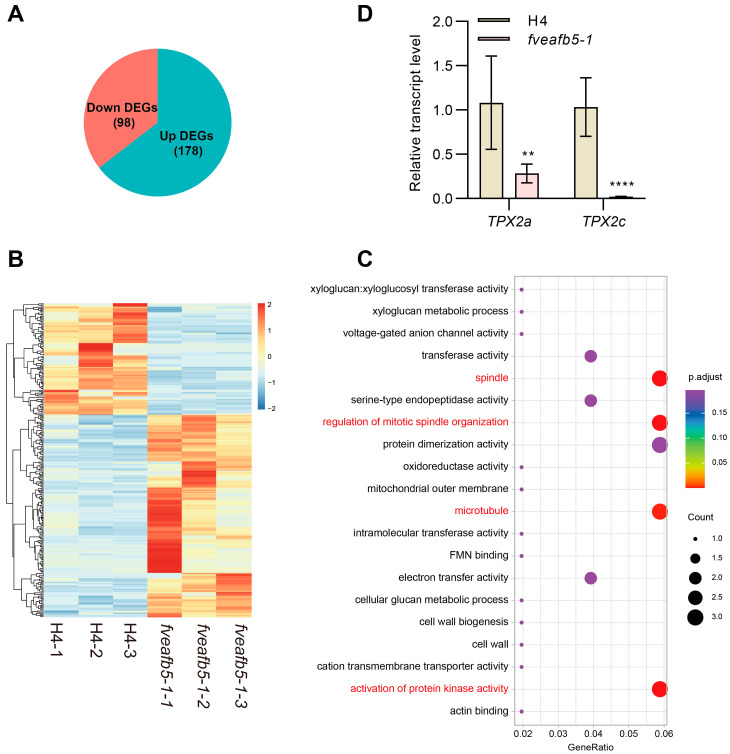
Transcriptome analysis of H4 and *fveafb5* during receptacle development. (**A**) The pie chart shows the differentially regulated genes (DEGs) at the S2 stage receptacle of the *fveafb5-1* mutant compared with H4. (**B**) Heat map analysis of the DEGs in H4 and the *fveafb5-1* mutant. (**C**) GO analysis of the down-regulated DEGs in stage II receptacle of the *fveafb5-1* mutant. (**D**) Quantitative reverse-transcription polymerase chain reaction (qRT-PCR) analyses show the relative transcript levels of the microtubule-associated gene *TPX2s* in the S2 stage receptacle in H4 and the *fveafb5-1* mutant. *FveACTIN* was used for normalization. One-way ANOVA and **, **** indicate significant differences at *p* < 0.01 and *p* < 0.0001, respectively (*n* = 3). At least three biological repeats showed a consistent, similar pattern.

Through transcriptome analysis of the H4 and *fveafb5* mutant at the S2 stage, we identified a total of 276 differentially expressed genes (DEGs). Among these DEGs, 178 were up-regulated, while 98 were down-regulated (Figure 3A). Heat map analysis revealed that the up-regulated and down-regulated DEGs clustered separately, signifying distinct expression patterns (Figure 3B). Gene ontology (GO) analysis of the downregulated DEGs demonstrated significant enrichment in the processes related to spindle, microtubules, and protein kinase activity (Figure 3C). For instance, *TPX2* (targeting protein for Xklp 2), a gene involved in regulating mitosis and strawberry organ size [34,35], exhibited significant down-regulation in the *fveafb5-1* mutant, as confirmed by quantitative RT-PCR (Figure 3D). Collectively, these results provide further evidence that FveAFB5 could be involved in strawberry fruit development.

### 2.4. FveAFB5 Mediates Auxin Responses in Strawberry Roots

**Figure 4:**
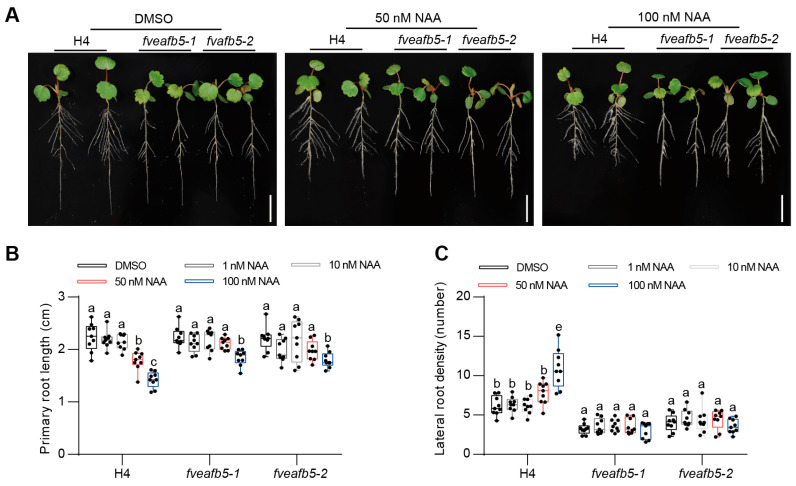
FveAFB5 mediated auxin regulation of primary root and lateral root development. (**A**) Root phenotype in H4 and *fveafb5* mutants under auxin treatment with different concentrations. (**B**,**C**) Quantification analysis of primary root length (**B**) and lateral root density (**C**). Scale bar: 1 cm. Two-way ANOVA; different letters represent significant differences at *p* < 0.0001 (*n* = 9). The experiments were repeated three times.

To investigate whether FveAFB5 is involved in mediating auxin responses in strawberries, we treated the *fveafb5* mutants with auxin and evaluated both primary root and lateral root phenotypes. The seedlings grew on a half-strength MS medium for 12 days and then were transferred to a half-strength MS medium containing different concentrations of auxin for 7 days. The application of exogenous auxin strongly inhibited primary root length and significantly promoted lateral root formation in the H4 wild type. However, in the *fveafb5* mutants, the effects of auxin on both primary root and lateral root were markedly reduced. Specifically, the primary roots in the mutants were longer, and the lateral root density was higher compared to the H4 wild type (Figure 4A–C). These findings suggest that auxin-regulated root development in the strawberry depends on FveAFB5. Furthermore, we examined the auxin responses in the *FveAFB5* overexpression lines. Interestingly, the overexpression of *FveAFB5* significantly enhanced auxin responses in strawberries, resulting in shorter primary root length and increased lateral root number compared to the H4 wild type (Figure S5A–C). Overall, these results indicate that *FveAFB5* plays a potential role in mediating auxin responses in strawberry roots.

### 2.5. FveAFB5 Mediates Auxinic Herbicides Responses in Strawberry

**Figure 5:**
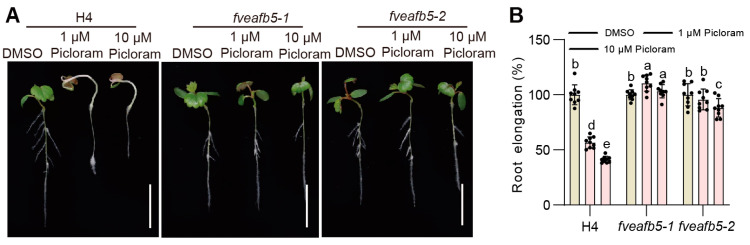
*FveAFB5* mutant seedlings show resistance to auxinic herbicide picloram. (**A**,**B**) Auxinic herbicide picloram resistance phenotype of *fveafb5* mutants. (**A**) H4 and *fveafb5* mutants treated with different concentrations of picloram for 5 days were observed. (**B**) Quantification analysis of root elongation phenotype in *fvafb5* mutants. Scale bar: 1 cm. Two-way ANOVA; different letters represent significant differences at *p* < 0.0001 (*n* = 9). Experiments were repeated three times.

**Figure 6:**
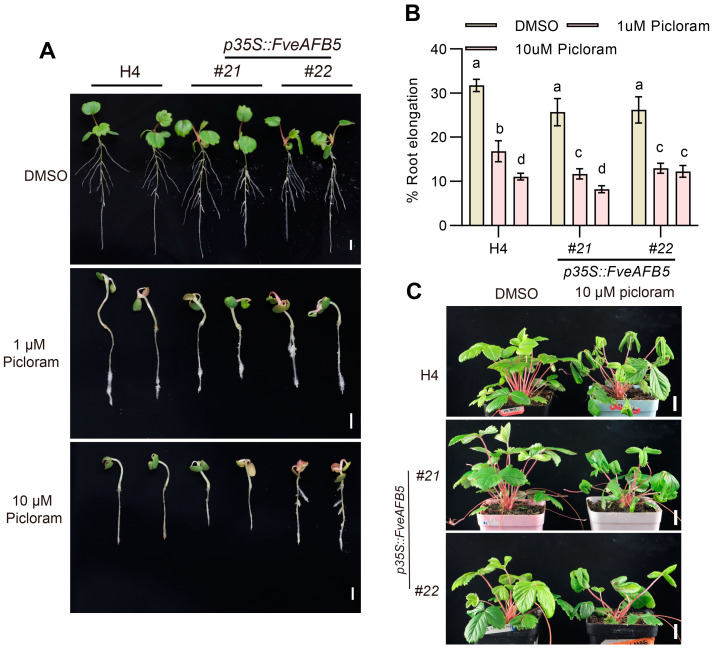
*FveAFB5* overexpression shows enhanced sensitivity to auxinic herbicides picloram. (**A**,**B**) Auxinic herbicide picloram resistance phenotype of *FveAFB5* overexpression transgenic lines. (**A**) H4 and *FveAFB5* overexpression transgenic lines treated with different picloram concentrations for 5 days were observed. (**B**) Quantification analysis of the root elongation phenotype. (**C**) Auxinic herbicide resistance phenotypes of 1-month-old H4 and *FveAFB5* overexpression transgenic lines treated with different concentrations of picloram for 10 days. Scale bar: 1 cm. Two-way ANOVA; different letters represent significant differences at *p* < 0.01 (*n* = 9). Experiments were repeated three times.

Synthetic auxin herbicides including phenoxy carboxylic acid (MCPA), benzoic acid (dicamba), pyridine carboxylic acid (picloram), and quinoline carboxylic acid (quinclorac) are extensively utilized in agriculture for their capacity to impede growth including disrupting plant physiology, inhibiting growth, and inducing geotropism at elevated concentrations [26,27]. To determine the involvement of FveAFB5 in auxinic herbicide responses in strawberries, we treated both the H4 wild type and the *fveafb5* mutants with the auxinic herbicides picloram, dicamba, and quinclorac, and observed their respective growth inhibition phenotypes. The herbicides are lethal at 2000 µM concentration, but the doses we used were low enough to show effects while being non-lethal. Interestingly, we observed that the *fveafb5* mutants exhibited normal growth under both 1 μM and 10 μM picloram treatments, in contrast to the strong growth inhibition observed in the H4 wild type (Figure 5). This suggests that the mutation in *FveAFB5* enhances resistance to picloram in strawberries. Furthermore, we observed a significant alleviation of the plant growth inhibition caused by dicamba and quinclorac in the *fveafb5* mutants (Figure S6), indicating that the mutants also displayed resistance to dicamba and quinclorac. Additionally, we explored the response of the *FveAFB5* overexpression lines to the auxinic herbicide picloram. Interestingly, overexpression of *FveAFB5* enhanced picloram responses, resulting in more severe growth inhibition compared to the H4 wild type (Figure 6). Overall, these results suggest that FveAFB5 plays a vital role in mediating auxinic herbicide responses in strawberries. This has inspired us to further investigate the downstream genes regulated by FveAFB5 in the context of auxinic herbicide responses.

**Figure 7:**
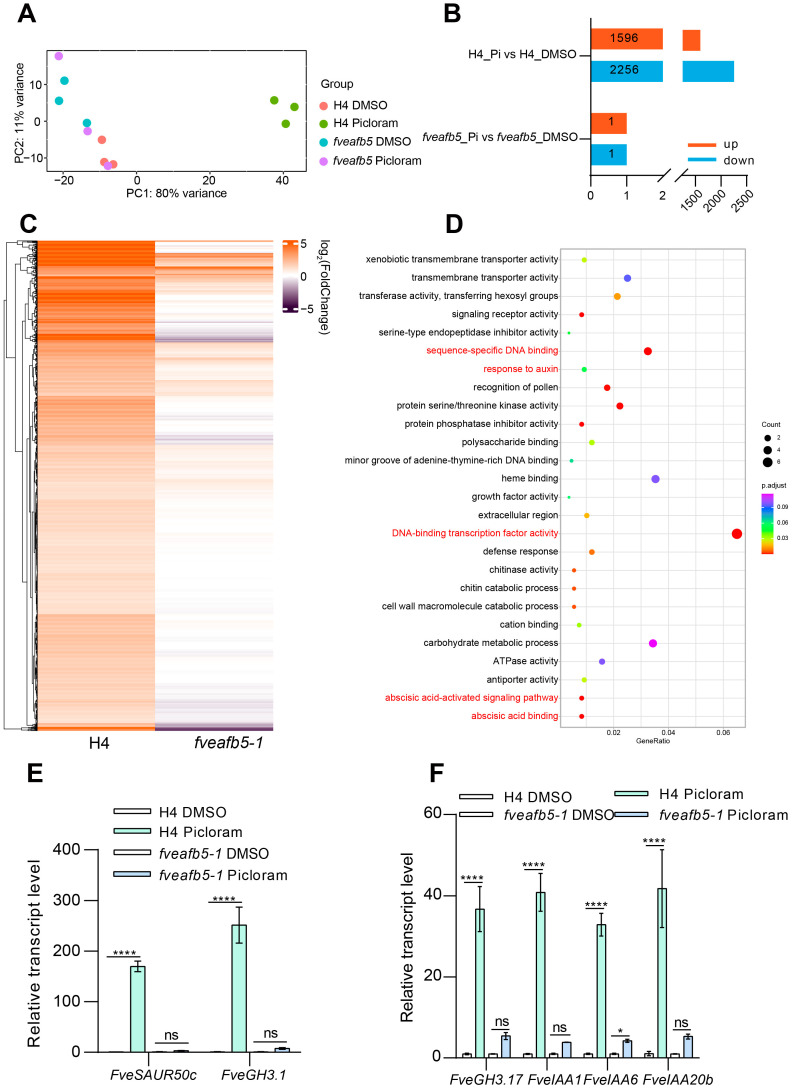
FveAFB5 mediates transcriptome reprogramming under picloram treatment. (**A**) PCA analysis of H4 and the *fveafb5-1* mutant with or without picloram treatment. (**B**) The column chart shows the number of differentially expressed genes (DEGs) of H4 and the *fveafb5-1* mutant treated with picloram. Up-regulated DEGs or down-regulated DEGs were marked with different colors. (**C**) The heat map shows the expression profile of the up-regulated DEGs in H4 and the *fveafb5-1* mutant after picloram treatment. (**D**) Gene ontology (GO) analysis shows the up-regulated DEGs in H4 and the *fveafb5-1* mutant after picloram treatment. GO analysis only shows the top 26 GO terms according to *q* value. The size of the pie chart area represents the number of enriched genes. DNA binding transcription factor, ABA, and auxin-related categories are marked with red characters. (**E**,**F**) Quantitative reverse transcription polymerase chain reaction (qRT-PCR) analyses show the relative transcript levels of auxin-related genes *FveSAUR/FveIAA/FveGH3* in H4 and the *fveafb5-1* mutant treated with 7 d picloram. *FveACTIN* was used for normalization. Two-way ANOVA and *, **** indicate significant differences at *p* < 0.05 and *p* < 0.0001, respectively; ns means no significance. At least three biological repeats showed a consistent, similar pattern.

Then we performed transcriptome analysis on wild-type (H4) seedlings and *fveafb5* mutant seedlings both treated with and without picloram. An intriguing finding emerged from the transcriptome analysis of the wild type under picloram treatment, revealing a distinct principal component analysis (PCA) plot compared to the relatively close PCA plot of the *fveafb5-1* mutant (Figure 7A), which aligns with the insensitive phenotype displayed by the *fveafb5* mutants under picloram treatment (Figure 5A,B). Subsequent analysis confirmed the presence of 3852 differentially expressed genes (DEGs) in H4 treated with picloram, with 1596 DEGs up-regulated and 2256 DEGs down-regulated (Figure 7B). In contrast, the *fveafb5* mutant exhibited only two DEGs, one up-regulated and one down-regulated (Figure 7B). Heat map analysis demonstrated the distinct clustering of up-regulated and down-regulated DEGs on separate branches (Figure 7C and Figure S7A). Gene ontology (GO) analysis of the FveAFB5-activated DEGs revealed their association with DNA-binding transcription factor activity, ABA, and auxin response-related processes (Figure 7D), while the FveAFB5-repressed DEGs were mainly involved in photosynthesis-related processes (Figure S7B). Furthermore, quantitative RT-PCR (qRT-PCR) validated that auxin-related genes were significantly induced by picloram treatment in H4, but the induction was partially abolished in the *fveafb5* mutant (Figure 7E,F). Overall, these findings suggest that under picloram treatment, FveAFB5 mediates the up-regulation of auxin and ABA-related genes as well as the down-regulation of photosynthesis-related genes. This activation of auxin and ABA signaling, coupled with the repression of photosynthesis, may be the cause of inhibited plant growth. In summary, the collective results presented in this study strongly indicate that FveAFB5 plays a crucial role in regulating auxinic herbicide responses in strawberries. By mediating transcriptome reprogramming, FveAFB5 influences the expression of a wide range of genes, leading to significant changes in key biological processes. This includes the up-regulation of auxin and ABA-related genes, as well as the down-regulation of photosynthesis-related genes, ultimately resulting in altered growth and development in response to auxinic herbicide treatment. These findings highlight the importance of FveAFB5 in modulating the response of strawberries to auxinic herbicides and shedding light on the underlying mechanisms of herbicide resistance.

In conclusion, our findings shed light on the pivotal role of the auxin receptor FveAFB5 in mediating auxin signaling during strawberry growth and fruit development. FveAFB5 acts as a key regulator of auxin signaling, influencing various aspects of strawberry plant growth, including lateral root development, plant height, and shoot branching. Moreover, we have demonstrated that FveAFB5 interacts with FveIAA proteins in vivo, highlighting its involvement in mediating auxinic herbicide responses. Overall, these discoveries provide a new and comprehensive understanding of how FveAFB5 mediates auxin signaling to regulate fundamental developmental processes and to influence the response to auxinic herbicides in strawberries.

## 3. Discussion

In plants, the response to auxin is dependent on the auxin receptors TIR1/AFB. Mutants in the *AtAFB4* gene in *Arabidopsis* exhibit various developmental defects, including reduced seed size and weight, shortened petioles and hypocotyls, decreased lateral roots, and stunted plant height [36]. Similarly, the *osafb4* mutant in rice displays decreased grain yield and plant height, and adventitious roots, as well as an increase in tillering number [28]. In addition to rice, the *psafb4/5* mutant in peas, also known as *rms2* (Strigolactones SL biosynthesis genes) shows a similar phenotype, including increased shoot branching, dwarf and bushy appearance, low expression of SL biosynthesis genes, and high auxin levels [37]. Interestingly, we have observed that the *fveafb5* mutant in strawberries exhibits analogous phenotypes to the *atafb4* mutant in *Arabidopsis*, the *osafb4* mutant in rice, and the *psafb4/5* in peas. These include reduced lateral root density and plant height, smaller fruit size, and increased shoot branching. 

According to the phenotype, there should be a potential link between auxin and strigolactones (SL). PsAFB4/5 plays a major and specific role not only in auxin and herbicide response but also in activating the strigolactone pathway as part of apical dominance and branch/tiller repression [37]. Hence, the *psafb4/5* mutants are a type of mutant specifically defective in strigolactones and show distinctively more branching/tillering than other *pstir1/afb* mutants. It has been suggested that picolinate herbicides may mimic a yet unidentified auxinic hormone [38,39]. The *psafb4/5* mutants appear similar to strigolactone mutants, which are dwarfish and bushy. However, they are not entirely the same as strigolactone mutants because apical auxin inhibits other hormones, such as cytokinins, which promote branching. Hence, *psafb4/5* mutants have both low strigolactones and high cytokinins but strigolactone mutants, such as *rms1*, have low cytokinins (and high auxin) [40]. Indeed, this is likely part of a strigolactone-PsAFB4/5 feedback system [39]. 

The regulation of fruit growth and development by the plant hormone auxin has been extensively studied in numerous species. For instance, auxin can induce parthenocarpy in tomatoes, regulate peach fruit size, control cell enlargement in cherries, and drive receptacle expansion in strawberries [41,42,43]. In our study, we demonstrate that the auxin receptor FveAFB5 is required for strawberry fruit development. Through transcriptome analysis, we have identified *FveTPX2s*, a family of microtubule-associated genes involved in mitosis and the regulation of organ size in strawberries, as potential downstream targets of FveAFB5. Further investigation is warranted to confirm the relationship between FveAFB5 and FveTPX2s during the process of strawberry fruit development. As further clarification, auxin is a major hormone that affects many traits that may have a compounding effect on adult plant phenotypes. Changes in fruit size and gene expression could be a direct effect but could equally be a consequence of dwarfing, high branching, or many other defects during earlier plant development. It would be worthwhile to further clarify the direct role of auxin in strawberry fruit development in future studies.

Herbicides play a crucial role in agricultural crop production, contributing to food security and improved yields. Among these, auxinic herbicides are commonly used to control dicot weeds and enhance modern crop production. However, the increasing resistance of weeds to auxinic herbicides has become a significant threat to agriculture [44]. The molecular mechanisms underlying herbicide resistance remain poorly understood. Previous studies in *Arabidopsis thaliana* and rice have demonstrated the essential role of the TIR1/AFB receptor in mediating responses to auxinic herbicides, with mutations in this receptor resulting in insensitivity to picloram [24,28]. 

Recent research revealed that 250 μM picloram alone was sufficient to produce mature woodland strawberry fruits to be as large as pollinated fruits [45]. In our research, we have shown that FveAFB5 is involved in the response to auxinic herbicides, as demonstrated by phenotypic and transcriptome analyses. Picloram, being a herbicide of low toxicity to humans and the environment, is widely used globally. Therefore, our findings have significant implications for the modification of effective herbicide-resistant genes and weed management in agronomically important crops. Considering the strong resistance of *fveafb5* mutants to picloram, it would be worthwhile to further investigate the structural features of FveAFB5 binding to different auxinic herbicides in future studies. 

## 4. Materials and Methods

### 4.1. Plant Materials and Growth Conditions

The woodland strawberry (*Fragaria vesca*) diploid variety Hawaii 4 (H4; white fruit) was selected as the genetic transformation target for stable strawberry transformation. *Nicotiana benthamiana* was chosen as the tobacco material for the experiments. To initiate the growth of strawberry seeds, a surface sterilization process was employed. First, the seeds were treated with 20% sodium hypochlorite for 10–15 min and washed with sterilized water. Second, the seeds were placed at 4 °C for a week. Then, the seeds were germinated on a half-strength Murashige and Skoog (MS) medium (Phytotechnology, Cat# M519, Overland Park, KS, USA) in a growth chamber (PERVICAL, Cat#AR-66L3, Perry, IA, USA). The growth chamber conditions were set at 23 °C ± 2 °C with a photoperiod of 16 h of light and 8 h of darkness. The humidity was maintained at 55%, and the illumination intensity was set to 100 µmol m^−2^ S^−1^.

### 4.2. Plasmid Construction and Tobacco Transformation

To create the *FveAFB5* CRISPR-Cas9 vector, two specific guide RNAs (sgRNAs) were designed to target the *FveAFB5* gene at positions 18 bp (sgRNA1: TCGTCGTCCCAAATGTCCGAGG) and 309 bp (sgRNA2: CGCTTCACCCGCGTCCGGGCGG) using the CRISPR-P web server available at http://crispr.hzau.edu.cn/CRISPR2/ (accessed on 14 April 2021). The sgRNA primers were synthesized, annealed, and inserted into the BbsI-digested AtU6-26 promoter to generate sgRNA cassettes. The two sgRNA cassettes were then digested with SpeI/SalI and NheI/SalI enzymes, respectively. This resulted in obtaining an sgRNA fragment and the plasmid skeleton, which were subsequently ligated together. Next, the tandem sgRNA cassette was digested with KpnI and SalI enzymes and then cloned into the *pUBQ10::Cas9-P2A-GFP* binary vector [46]. The resulting vector contains the Cas9 gene under the control of the ubiquitin 10 promoter (*pUBQ10*), *P2A-GFP* sequence, and the tandem sgRNA cassette for *FveAFB5* targeting.

To generate the *FveAFB5* overexpression vector, the coding sequence of *FveAFB5* was amplified through a polymerase chain reaction (PCR) and then introduced into the pK7WG2D.1 binary vector [35] using a gateway reaction. This allows for the expression of *FveAFB5* under the control of the appropriate promoter in the resulting pK7WG2D.1-FveAFB5 vector. For subcellular localization vectors, the coding sequence of *FveAFB5* was PCR-amplified and cloned into the pGWB505-GFP binary vector [47] using a gateway reaction. This enabled the fusion of the FveAFB5 protein with a green fluorescent protein (GFP) for subcellular localization studies. To construct the BiFC vectors, the coding sequences of *FveAFB5* and *FveIAA4a*, *FveIAA8b*, *FveIAA11*, *FveIAA17*, *FveIAA20*, and *FveIAA27a* were cloned into the p2YC and p2YN vectors [48]. This resulted in the generation of p2YC-FveAFB5 and p2YN-FveIAA4a/FveIAA8b/FveIAA11/FveIAA17/FveIAA20/FveIAA27a vectors respectively for bimolecular fluorescence complementation (BiFC) experiments.

For the subcellular localization experiment, the pGWB505-FveAFB5 vector and the p2YC-FveAFB5, p2YN-FveIAA4a/FvIAA8b/FveIAA11/FveIAA17/FveIAA20/FveIAA27a vectors were separately introduced into the *Agrobacterium* strain GV3101. The transformed GV3101 cells were then cultured on an LB solid medium overnight at 28 °C, followed by shaking in 10 mL of liquid medium until reaching an optical density (OD) of 0.6.

The *Agrobacterium* cells in the liquid medium were harvested by centrifugation at 4000 rpm for 10 min. The pellet was resuspended in suspension buffer (10 mM MES, pH 5.7, 10 mM MgCl_2_, 200 μM Acetosyringone (AS)) to achieve an OD 600 of 0.6. The suspension was then left at room temperature in the dark for 2–3 h to allow the *Agrobacterium* cells to recover. Next, approximately one-month-old *Nicotina benthamiana* leaves were selected for injection. The *Agrobacterium* suspension was injected into the chosen *N. benthamiana* leaves, causing a water-soaked appearance at the injection sites. The injected leaves were marked for identification. The injected tobacco plants were placed in a greenhouse and kept in darkness for 18–24 h to allow time for bacterial growth and expression. Subsequently, the GFP or YFP signal was observed using a Leica SP8 confocal microscope (Leica TCS SP8X DLS, excitation: 514 nm, emission: 520–550 nm, Wetzlar, Germany). These experiments allowed for the visualization and examination of the subcellular localization of FveAFB5 and the interaction between FveAFB5 and various FveIAA proteins in *Nicotiana benthamiana*. The experiments were repeated three times.

Supplementary Table S1 contains a list of all the primers used in this process.

### 4.3. Strawberry Transformation

*Agrobacterium*-mediated strawberry transformation was carried out as follows: Newly unfolded leaves from the *F. vesca* Hawaii 4 (1–2 month tissue cultured seedlings) were collected and 2–3 transverse cuts in the direction relative to the main vein of the leaves were made using a scalpel. Then we placed the leaves into an *Agrobacterium* suspension which was diluted with infection solution (2.22 g/L MS (Phytotechnology, Cat#M404, KS, USA), 0.2% sucrose, 4 g/L MES (Sigma, Cat#M3671, St. Louis, MO, USA), 2 mg/L ZT (Yuanye, Cat#S18151, Shanghai, China), 80 mg/L Acetosyringone (Sigma, Cat#D1344065G, MO, USA), and 150 mg/L DTT (Sigma, Cat#43815, MO, USA), pH 5.4) to an OD_600_ of 0.6, and the leaves were soaked for 20 min. We dried the leaves on sterile filter paper to remove excess bacterial suspension. We transferred the leaves onto a co-cultivation medium (2.22 g/L MS, 0.2% sucrose, 4 g/L MES, 2 mg/L ZT, 80 mg/L Acetosyringone, 150 mg/L DTT, 8 g/L Agar (Phytotechnology, Cat#A296, USA), pH 5.4) covered with filter paper. We incubated them for 3 days in the dark at 23 °C. The leaves were then transferred to a recovery medium (4.43 g/L MS, 0.2% sucrose, 0.6 g/L MES, 4 mg/L TDZ (BBI, Cat#51707, Shanghai, China), 0.1 mg/L IBA (Sigma, Cat#I5386, MO, USA), 250 mg/L Cefotaxime (Kulaibo, Cat#CC3251, Beijing, China), 250 mg/L Timentin (Suolaibao, Cat#T8660, Beijing, China), and 7 g/L Agar, pH 5.6) and incubated them in the dark for 2–4 weeks until the calli formed. Subsequently, the calli were transferred to a screening medium containing 5 mg/L kanamycin (Kulaibo, Cat#CK6731, Beijing, China) or 1 mg/L hygromycin B (YEASEN, Cat# 60224ES03, Shanghai, China) for 2 weeks. Then the screened leaves were transformed to the screening medium containing 15 mg/L kanamycin or 2 mg/L hygromycin for another two weeks. At last, the leaves were transformed to the screening medium containing up to 20 mg/L kanamycin or 3 mg/L hygromycin for further screening, and meanwhile TDZ was changed to 3 mg/L 6-BA (Sigma, Cat#B3408, MO, USA), until the calli grew green buds. The buds were transferred to a differentiation medium (3.21g/L B5 (Phytotechnology, Cat#G398, USA), 0.2% sucrose, 0.6 g/L MES, 0.5 mg/L 6-BA, 0.1 mg/L IBA, 250 mg/L Cefotaxime, 250 mg/L Timentin, 7.5 g/L Agar, pH 5.6). We then performed sub-culturing every two weeks to a month until seedlings were generated. The seedlings were transferred to a half-strength MS medium (2.22 g/L MS, 0.2% sucrose, 0.6 g/L MES, 7 g/L Agar pH 5.6). After one month of growth, we extracted DNA from the young seedlings and performed genotyping to confirm the presence of the hygromycin/kanamycin resistance gene. 

Supplementary Table S1 contains a list of all the primers used in this process.

### 4.4. Auxin Treatment and Phenotype Observation

We chose fully opened flowers from two-month-old strawberries grown in soil to conduct artificial pollination. After pollination, normal fruits were selected for late phenotype observation and measurement. We used 10–20 fruits in each treatment and measured fruit width and height using a vernier caliper.

For seedling auxin treatment, sterilized seeds were first grown on a half-strength MS medium for 12 days. The seedlings were then transferred to a half-strength MS medium containing different concentrations of NAA (Sigma, Cat#N064, USA) for 7 days, and the root phenotypes were measured using ImageJ software (Fiji, https://imagej.net/ij/).

For seedling auxinic herbicide treatment, sterilized seeds were grown on a half-strength MS medium for 12 days. The seedlings were then transferred to a half-strength MS medium containing different concentrations of picloram (Sangon, Cat#A610733, Shanghai, China), dicamba (Sangon, Cat#A600393, Shanghai, China), and quinclorac (OKA, Cat#84087, Beijing, China) for 7 days. The root phenotypes were calculated using ImageJ software.

For large seedling auxinic herbicide treatment, sterilized seeds were grown on a half-strength MS medium for 21 days before being transferred to soil. After one month of growth, the seedlings were sprayed with various concentrations of picloram (1 μM and 10 μM), dicamba (10 μM and 50 μM), and quinclorac (50 μM and 100 μM). The spray was repeated every two days, and the phenotypes were observed.

These procedures were conducted to assess the effects of auxin and auxinic herbicides on fruit development, seedling root phenotypes, and larger seedling growth.

### 4.5. Gene Expression Analysis

For gene expression analysis, total RNA was extracted using the Total RNA Extraction Kit of Polysaccharide Polyphenol (TianGen, Cat#DP441, Beijing, China) following the instructions provided with the kit.

One microgram of total RNA was reverse-transcribed using Moloney-Murine Leukemia Virus Reverse Transcriptase (Promega, Cat#M314C, Madison, WI, USA). The resulting complementary DNA (cDNA) was then used for quantitative reverse transcription polymerase chain reaction (qRT-PCR). Half a percent of the cDNA was used as the template for qRT-PCR analysis.

The housekeeping gene *FveACTIN* was used as an endogenous control for qRT-PCR normalization. The relative expression level of each gene was calculated using the 2^−∆∆Ct^ (cycle threshold) method [49]. All qRT-PCR analyses were performed with three independent biological replicates.

A list of all the primers used can be found in Table S1.

### 4.6. Messenger RNA Sequencing and Transcriptome Data Analysis 

For mRNA sequencing of strawberry fruit, young fruits from the H4 wild type and *fveafb5-2* mutant were selected at the S2 fruit development stage, which corresponds to 2–4 days after pollination. Approximately 0.3 g of fruit material, equivalent to around 20 fruits, was quickly frozen in liquid nitrogen to preserve the RNA.

For mRNA sequencing of seedlings treated with picloram, the seeds of both H4 and the *fveafb5-2* mutant were subjected to surface sterilization using a 20% sodium hypochlorite solution. After one week of vernalization at 4 °C, the seeds were germinated vertically on a half-strength MS medium for 12 days. Following this, the seedlings with consistent growth conditions were transferred to a half-strength MS medium containing either DMSO (control) or 1 μM picloram for an additional 5 days of vertical growth.

The materials, including fruit and treated seedlings, were harvested, and total RNA was isolated using the same protocol described earlier. The sequencing was performed using the Illumina Nova Seq6000 platform (150-bp paired-end reads) in Novogene, Beijing, China, with three independent biological replicates for each condition; 6G sequencing depth was performed for each sample.

For the transcriptome analysis, the following steps and tools were utilized. (1) Reference Genome Comparison: The strawberry reference genome sequence and gene annotation file were downloaded from the strawberry genome database *Fragaria vesca* Genome v4.0.a2 (https://www.rosaceae.org/species/fragaria_vesca/genome_v4.0.a2 (accessed on 10 February 2022)). (2) Sequence Alignment: The R language in a Linux environment was used for sequencing sequence alignment. The alignment was performed using the reference genome sequence. (3) Gene Function Annotation: The strawberry protein annotation and transcriptome annotation from the *Fragaria vesca* database (https://www.rosaceae.org/species/fragaria/all (accessed on 1 December 2022)) were used for gene function annotation in the sequencing data. (4) Gene Expression Analysis: The number of mapped reads and transcript length in the samples were normalized. Counts per million (CPM) standardization was used as an indicator of the gene expression level. Correlation analysis, cluster analysis, and principal component analysis (PCA) were performed using log2(fold change) ≥ 1 and *padj* < 0.05 as the criteria. DESeq^2^ was used to screen differentially expressed genes among the sample groups (http://www.biocloud.net/gongju (accessed on 15 December 2022)). (5) GO Functional Annotation: The strawberry GO annotation database Fvevesca_v4.0.a2_genes2GO (https://www.rosaceae.org/species/fragaria_vesca/genome_v4.0.a2 (accessed on 20 December 2022)) was used for GO functional annotation of the gene sequencing data. (6) Heatmap Analysis: The BMKCloud website (http://www.biocloud.net/gongju (accessed on 23 December 2022)) was used for the screening of differentially expressed genes for heatmap analysis. (7) Phylogenetic Tree Construction: The TAIR website (https://www.arabidopsis.org/ (accessed on 25 March 2022)) was utilized to find protein sequences in *Arabidopsis thaliana*. NCBI BLAST (https://blast.ncbi.nlm.nih.gov/Blast.cgi (accessed on 26 March 2022)) and Phytozome (https://phytozome-next.jgi.doe.gov/ (accessed on 28 March 2022)) were used to obtain homologous genes of the strawberry. Clustal W was used for multi-sequence alignment, and MEGA5.0 software (https://sourceforge.net/projects/mega5/ (accessed on 29 March 2022)) was applied to construct a phylogenetic tree using the neighbor-joining method with bootstrap analysis (500 repeats). These tools and methods were employed to analyze and annotate the transcriptome data, identify differentially expressed genes, perform functional annotation, and construct phylogenetic trees in the study of strawberry gene expression analysis.

## Data Availability

All relevant data are included within the article and its Supplementary Materials.

## Supplementary Materials

The following supporting information can be downloaded at: https://www.mdpi.com/article/10.3390/plants13081142/s1. Figure S1. Phylogenetic tree and protein domain analysis of AtTIR1/AFB and FveTIR1/AFB. (A) Maximum likelihood tree of AtTIR1/AFB and its homologs FveTIR1/AFB. Numbers on each branch represent the corresponding bootstrap probability values obtained in 500 replications. Proteins marked with red color represent FveTIR1/AFB proteins. (B) Amino acid sequence alignment of FveTIR1/AFB and AtTIR1/AFB proteins. F-box and LRR domains were indicated respectively. Figure S2. Expression pattern and subcellular localization of FveAFB5. (A,B) Quantitative reverse-transcription polymerase chain reaction (qRT-PCR) analyses (A) and eFP gene expression heatmap (https://bar.utoronto.ca/efp_strawberry/cgi-bin/efpWeb.cgi (accessed on 10 March 2022)) (B) of FveAFB5 in the different tissues of strawberries. (C) Subcellular localization of FveAFB5-GFP fusion protein in Nicotina benthamiana leaf epidermis cells. p35S::GFP acts as negative control. Scale bar, 50 μm. Figure S3. FveAFB5 interacts with FveIAA proteins in vivo. The interaction between FveAFB5 and FveIAA proteins was determined by bimolecular fluorescence complementation (BiFC) imaging assays in Nicotiana benthamiana leaves. nYFP, N-terminal region of bimolecular fluorescence; cYFP, C-terminal region of bimolecular fluorescence. Scale bar, 100 μm. Figure S4. FveAFB5 overexpression leads to more lateral root. (A–C) Identification of FveAFB5 overexpression transgenic plants. (A) Main components of FveAFB5 overexpression vector pK7WG2D.1-FveAFB5. (B) PCR verification of FveAFB5 overexpression transgenic plants, “+” represents the vector as a positive control, and “-” represents H4 as a negative control. (C) qRT-PCR identifies FveAFB5 gene expression in the FveAFB5 overexpression transgenic plants. (D,E) Root phenotype (D) and quantification analysis of the lateral root density (E) in FveAFB5 overexpression transgenic plants. One-way ANOVA, * represent significant difference at *p* < 0.05 (*n* = 9). Scale bars, 1 cm. The experiments were repeated at least 3 times and showed similar, consistent results. Figure S5. FveAFB5 overexpression shows hypersensitive to auxin during primary root and lateral root development. (A) Root phenotype in H4 and FveAFB5 overexpression lines under auxin treatment with different concentrations. (B,C) Quantification analysis of the primary root length (B) and lateral root density (C). Scale bar, 1 cm. Two-way ANOVA, different letters represent significant difference at *p* < 0.01 (*n* = 9). Figure S6. FveAFB5 mutation shows resistance to auxinic herbicides dicamba and quinclorac. (A–C) Resistance phenotype of *fveafb5* mutants to 1 μM or 10 μM auxinic herbicides dicamba and quinclorac. (A) H4 and *fveafb5* mutants treated with 1 μM or 10 μM dicamba and quinclorac concentrations for 5 days were observed. (B,C) Quantification analysis of the root elongation phenotype under dicamba (B) and quinclorac (C) treatment respectively. (D–F) The resistance phenotype of *fveafb5* mutants treated with 10 μM or 50 μM auxinic herbicides dicamba and quinclorac. (D) H4 and *fveafb5* mutants treated with 10 μM or 50 μM dicamba and quinclorac concentrations for 5 days were observed. (E,F) Quantification analysis of the root elongation phenotype under dicamba (E) and quinclorac (F) treatment respectively. Scale bar, 1 cm and two-way ANOVA, different letters represent significant difference at *p* < 0.01 (*n* = 9). The experiments were repeated at least 3 times and showed similar, consistent results. (B,C) Quantification analysis of the primary root length (B) and lateral root density (C). Scale bar, 1 cm. Two-way ANOVA, different letters represent significant difference at *p* < 0.01 (*n* = 9). Figure S7. FveAFB5 mediates transcriptome reprogramming under auxinic herbicides picloram treatment. (A) Heatmap shows the different fold changes of the down-regulated DEGs in H4 and *fveafb5-1* mutant under picloram treatment. (B) Gene Ontology (GO) analysis shows the down-regulated DEGs in the H4 and *fveafb5-1* mu-tant under picloram treatment. GO analysis only shows the top 19 GO terms according to q value. The size of the pie chart area represents the number of enriched genes. Photosynthesis-related categories were marked by red characters. Table S1. List of primers used in this study. Table S2. List of up- and down-regulated genes at stage 2 fruit development stage in *fveafb5* compared with H4. Table S3. List of up- and down-regulated genes after 5-day picloram treatment in H4. Table S4. List of up- and down-regulated genes after 5-day picloram treatment in *fveafb5*. Table S5. GO enrichment analysis of FveAFB5-activated up-regulation DEGs at stage 2 fruit development stage. Table S6. GO enrichment analysis of FveAFB5-activated up-regulation DEGs after 5-day picloram treatment. Table S7. GO enrichment analysis of FveAFB5-repressed down-regulation DEGs after 5-day picloram treatment.
