# Supplementary material for: The Role of FveAFB5 in Auxin-Mediated Responses and Growth in Strawberries
Source: Plants (Basel). 2024 Apr 19;13(8):1142. doi: 10.3390/plants13081142 (PMC11055006; doi:10.3390/plants13081142)
Supplement: Supplementary file 1 [file plants-13-01142-s001.zip › plants-2930593-supplementary.pdf]

plants

Supplementary Materials for

The Role of FveAFB5 in Auxin-Mediated Responses and Growth in Strawberries

Xuhui Wang [1,2], Shuo Feng [2], Jiangshan Luo [2], Shikui Song [2], Juncheng Lin [2], Yunhe Tian [2], Tongda Xu [2,*] and Jun Ma [2,*]

This file includes:

Supplementary Figures S1 to S7

Supplementary Table Sets

Supplementary figures

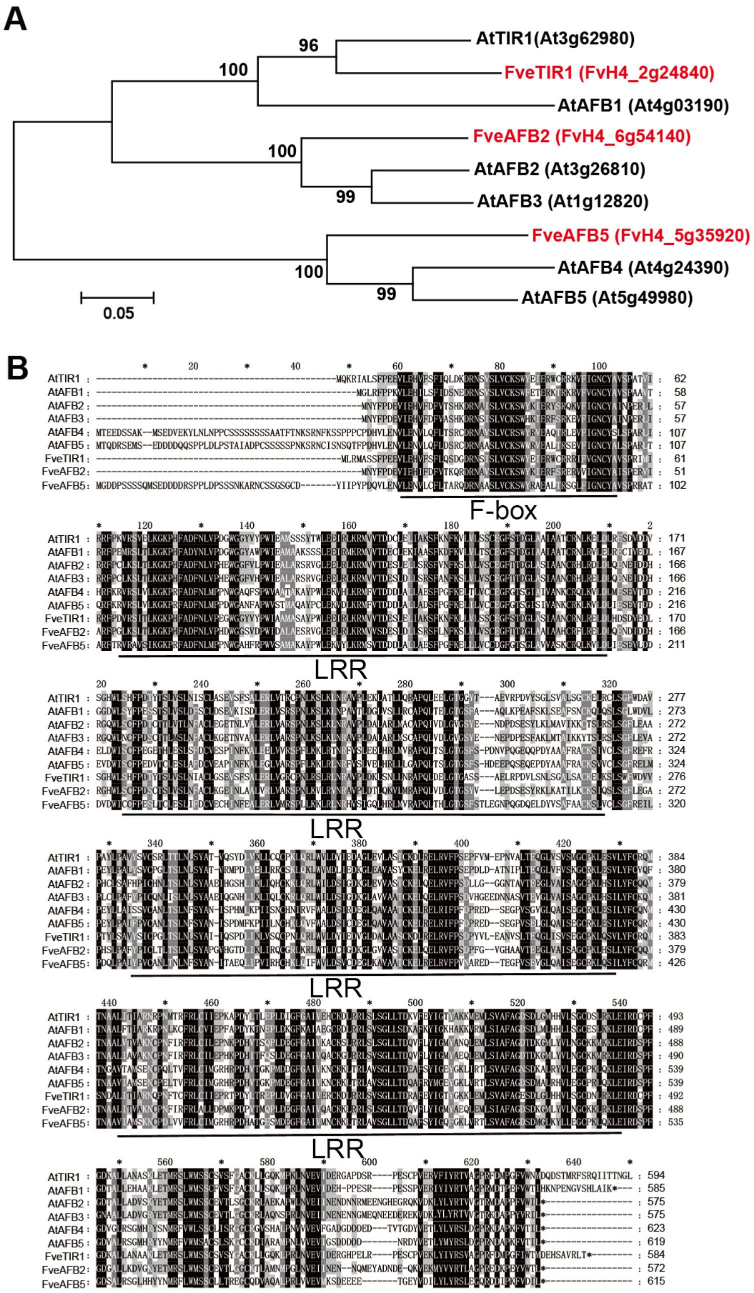

Figure S1. Phylogenetic tree and protein domain analysis of AtTIR1/AFB and FveTIR1/AFB.

(**A**) Maximum likelihood tree of AtTIR1/AFB and its homologs FveTIR1/AFB. Numbers on each branch represent the corresponding bootstrap probability values obtained in 500 replications. Proteins marked with red color represent FveTIR1/AFB proteins.

(**B**) Amino acid sequence alignment of FveTIR1/AFB and AtTIR1/AFB proteins. F-box and LRR domains were indicated respectively.

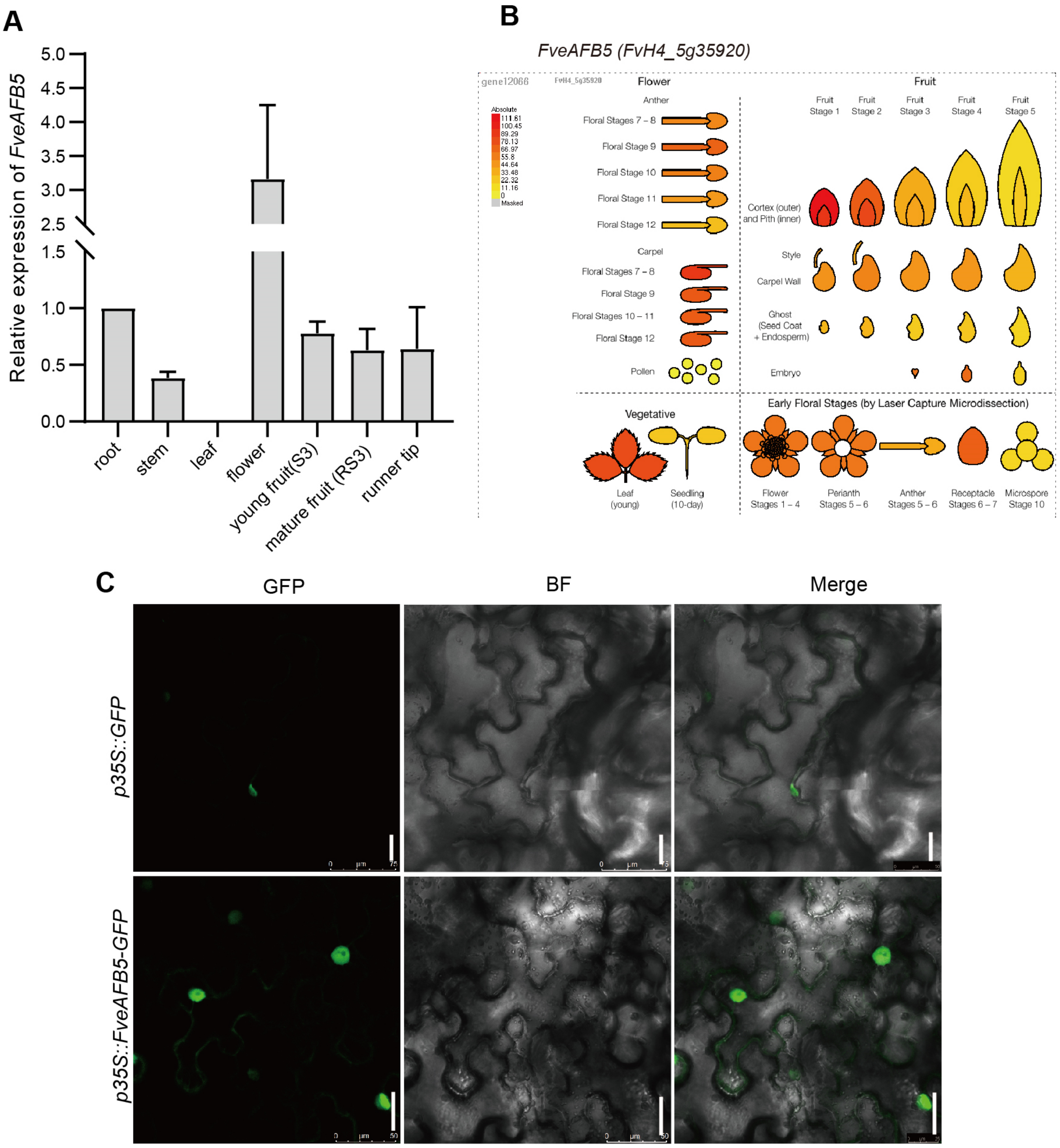

Figure S2. Expression pattern and subcellular localization of FveAFB5.

(**A**,**B**) Quantitative reverse-transcription polymerase chain reaction (qRT-PCR) analyses (**A**) and eFP gene expression heatmap (https://bar.utoronto.ca/efp_strawberry/cgi-bin/efpWeb.cgi (accessed on 10 March 2022)) (**B**) of *FveAFB5* in the different tissues of strawberries.

(**C**) Subcellular localization of FveAFB5-GFP fusion protein in *Nicotina benthamiana* leaf epidermis cells. *p35S::GFP* acts as negative control. Scale bar, 50 μm.

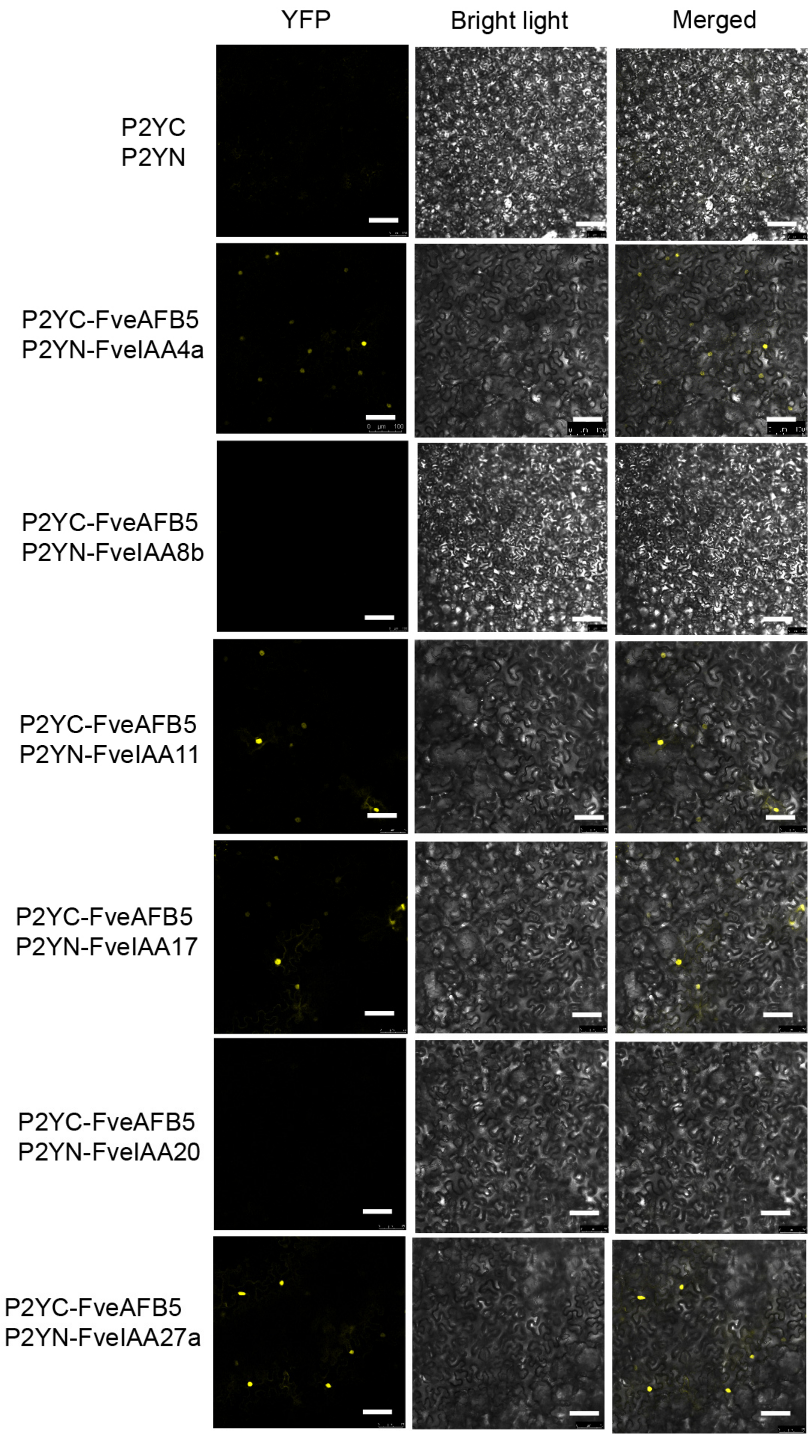

Figure S3. FveAFB5 interacts with FveIAA proteins in vivo.

The interaction between FveAFB5 and FveIAA proteins was determined by bimolecular fluorescence complementation (BiFC) imaging assays in *Nicotiana benthamiana* leaves. nYFP, N-terminal region of bimolecular fluorescence; cYFP, C-terminal region of bimolecular fluorescence. Scale bar, 100 μm.

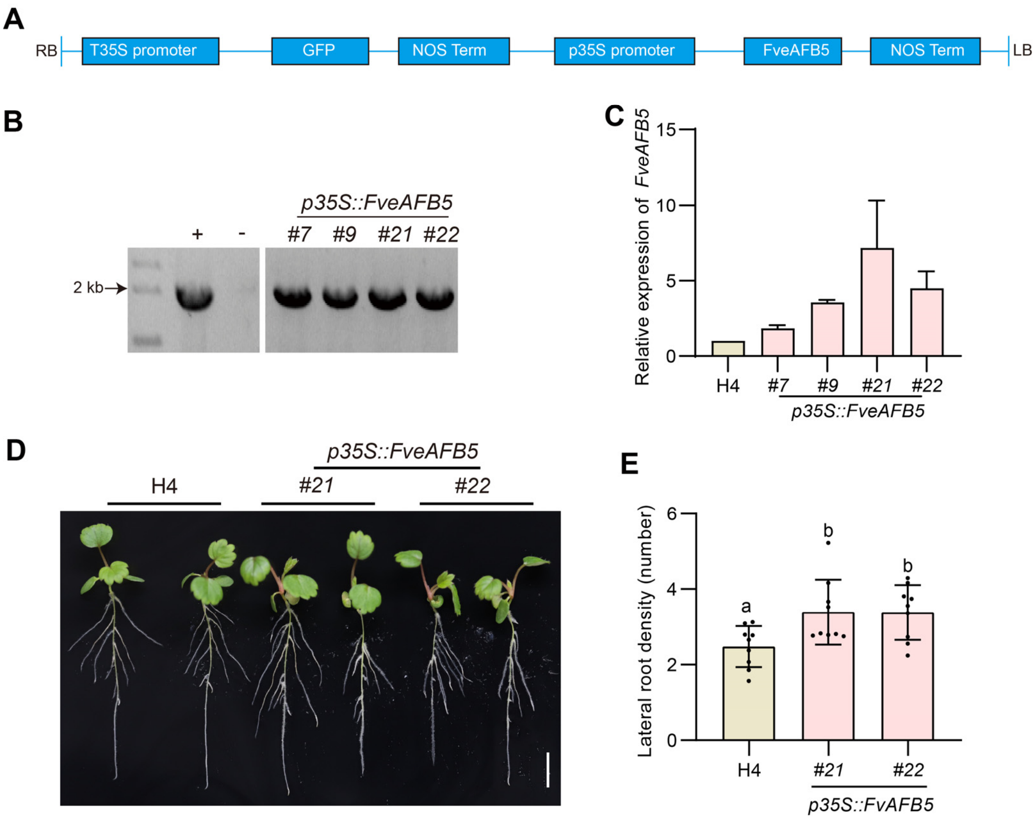

Figure S4. *FveAFB5* overexpression leads to more lateral root.

(**A–C**) Identification of *FveAFB5* overexpression transgenic plants. (**A**) Main components of *FveAFB5* overexpression vector pK7WG2D.1-FveAFB5. (**B**) PCR verification of *FveAFB5* overexpression transgenic plants, "+" represents the vector as a positive control, and "-" represents H4 as a negative control. (**C**) qRT-PCR identifies *FveAFB5* gene expression in the *FveAFB5* overexpression transgenic plants.

(**D,E**) Root phenotype (**D**) and quantification analysis of the lateral root density (**E**) in *FveAFB5* overexpression transgenic plants. One-way ANOVA, * represent significant difference at $P < 0.05$ ($n = 9$). Scale bars, 1 cm. The experiments were repeated at least 3 times and showed similar, consistent results.

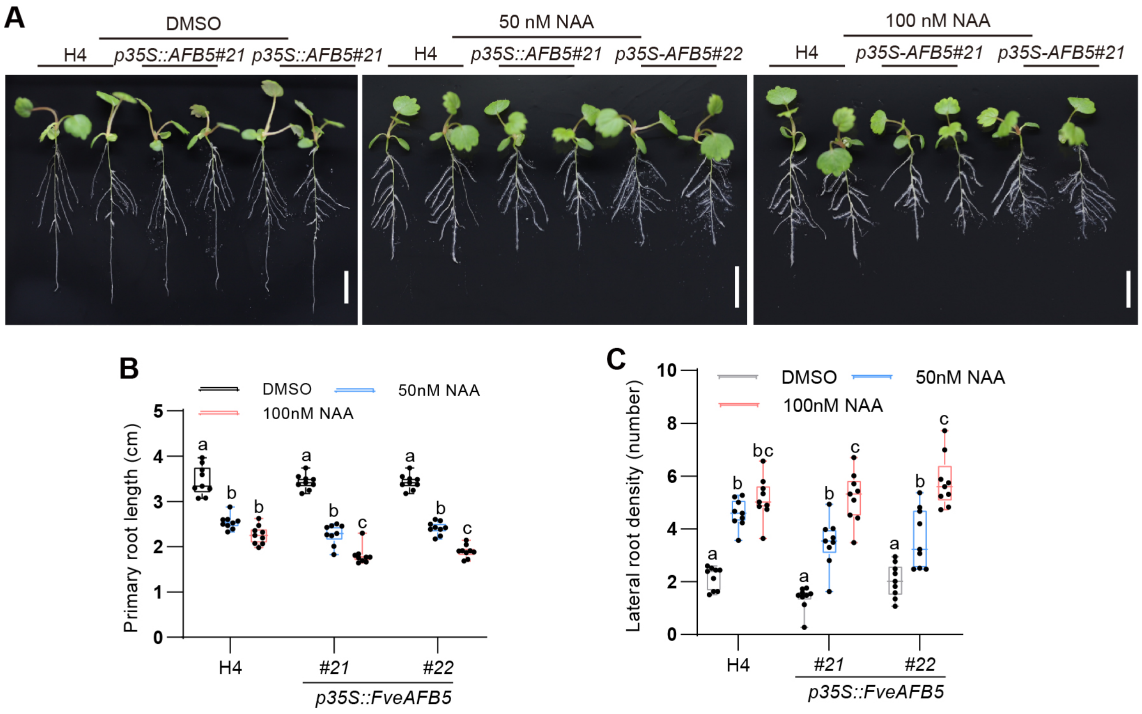

Figure S5. *FveAFB5* overexpression shows hypersensitive to auxin during primary root and lateral root development (**A**) Root phenotype in H4 and *FveAFB5* overexpression lines under auxin treatment with different concentrations. (**B**,**C**) Quantification analysis of the primary root length (**B**) and lateral root density (**C**). Scale bar, 1 cm. Two-way ANOVA, different letters represent significant difference at $P < 0.01$ ($n = 9$).

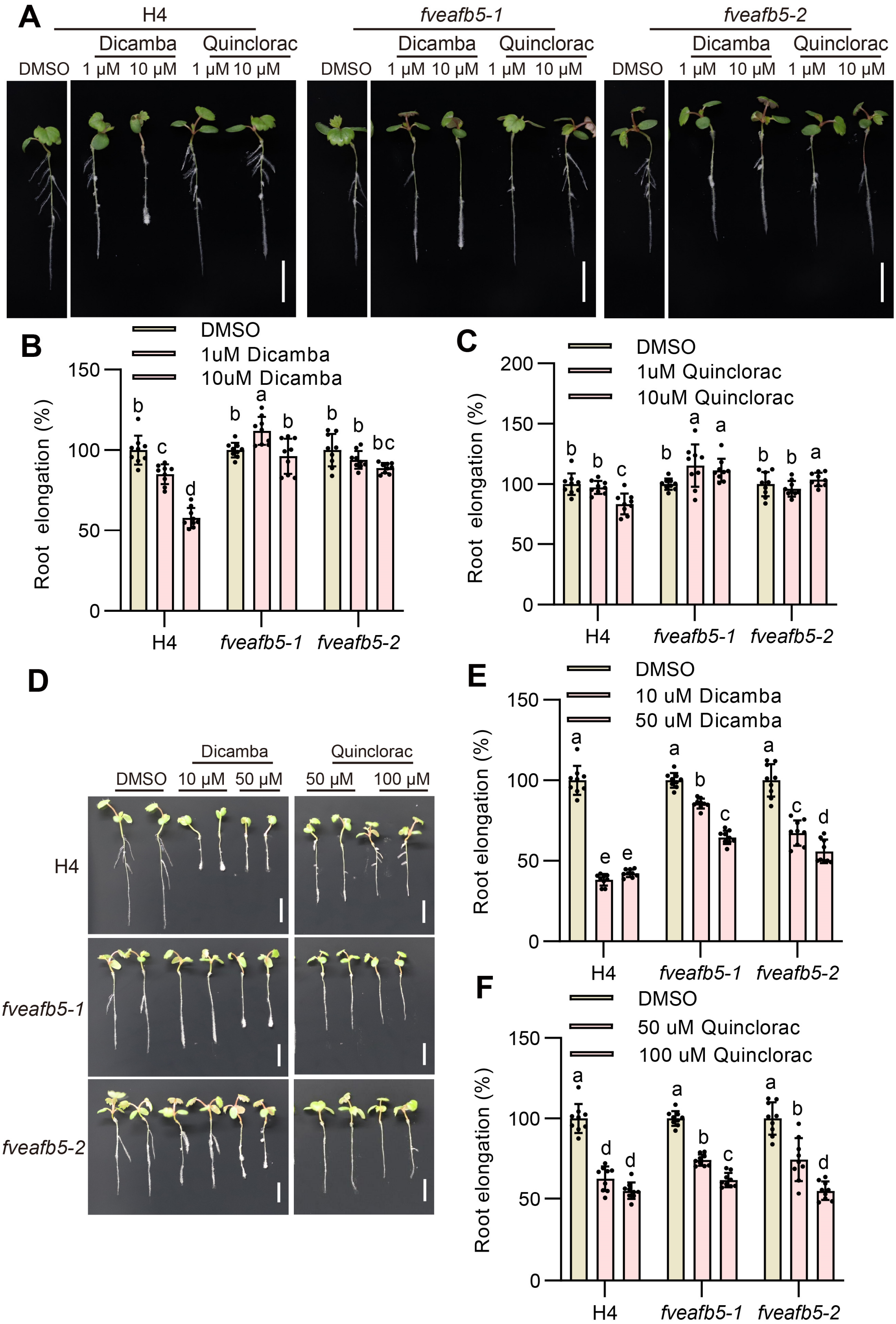

Figure S6. *FveAFB5* mutation shows resistance to auxinic herbicides dicamba and quinclorac.

(**A–C**) Resistance phenotype of *fveafb5* mutants to 1 μM or 10 μM auxinic herbicides dicamba and quinclorac. (**A**) H4 and *fveafb5* mutants treated with 1 μM or 10 μM dicamba and quinclorac concentrations for 5 days were observed. (**B-C**) Quantification analysis of the root elongation phenotype under dicamba (**B**) and quinclorac (**C**) treatment respectively.

(**D–F**) The resistance phenotype of *fveafb5* mutants treated with 10 μM or 50 μM auxinic herbicides dicamba and quinclorac. (**D**) H4 and *fveafb5* mutants treated with 10 μM or 50 μM dicamba and quinclorac concentrations for 5 days were observed. (**E,F**) Quantification analysis of the root elongation phenotype under dicamba (**E**) and quinclorac (**F**) treatment respectively. Scale bar, 1 cm and two-way ANOVA, different letters represent significant difference at $P < 0.01$ ($n = 9$). The experiments were repeated at least 3 times and showed similar, consistent results.

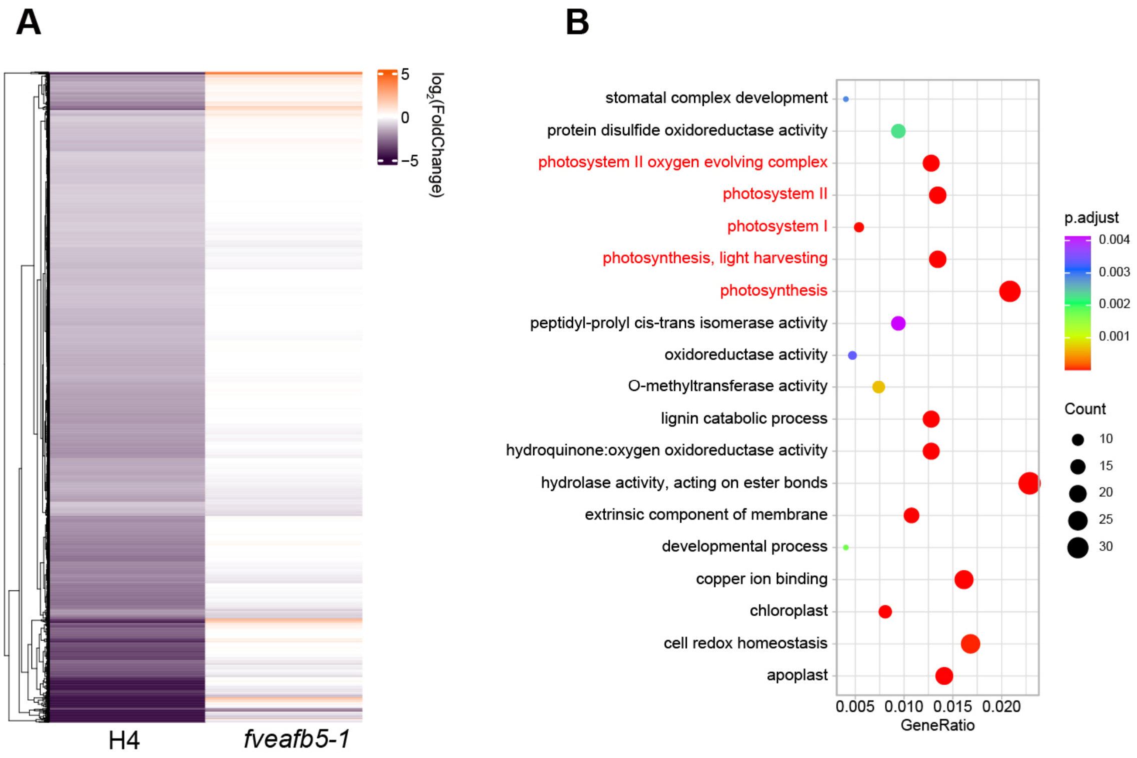

Figure S7. FveAFB5 mediates transcriptome reprogramming under auxinic herbicides picloram treatment

(**A**) Heatmap shows the different fold changes of the down-regulated DEGs in H4 and *fveafb5-1* mutant under picloram treatment. (**B**) Gene Ontology (GO) analysis shows the down-regulated DEGs in the H4 and *fveafb5-1* mutant under picloram treatment. GO analysis only shows the top 19 GO terms according to *q* value. The size of the pie chart area represents the number of enriched genes. Photosynthesis-related categories were marked by red characters.

Supplementary Table Sets

Supplementary Table S1. List of primers used in this study.

All primers used for genotyping, generation of the constructs and qRT-PCR are listed. The purposes of these primers are listed in the left. The name and sequence of these primers are displayed in the right. F, forward; R, reverse.

Supplementary Table S2. List of up- and down-regulated genes at stage 2 fruit development stage in *fveafb5* compared with H4.

Data includes both up- and down-regulated genes at stage 2 fruit development stage in *fveafb5* compared with in H4. Differentially expressed genes (DEGs) (padj < 0.05, CPM > 1, $Log_2FC > 1$ for up-regulated and $Log_2FC < -1$ for down-regulated) were normalized and extracted.

Supplementary Table S3. List of up- and down-regulated genes after 5-day picloram treatment in H4.

Data includes both up- and down-regulated genes after 5-day picloram treatment compared with blank treatment in H4. Differentially expressed genes (DEGs) (padj < 0.05, CPM > 1, $Log_2FC > 1$ for up-regulated and $Log_2FC < -1$ for down-regulated) were normalized and extracted.

Supplementary Table S4. List of up- and down-regulated genes after 5-day picloram treatment in *fveafb5*.

Data includes both up- and down-regulated genes after 5-day picloram treatment compared with blank treatment in *fveafb5*. Differentially expressed genes (DEGs) (padj < 0.05, CPM > 1, $Log_2FC > 1$ for up-regulated and $Log_2FC < -1$ for down-regulated) were normalized and extracted.

Supplementary Table S5. GO enrichment analysis of FveAFB5-activated up-regulation DEGs at stage 2 fruit development stage.

Supplementary Table S6. GO enrichment analysis of FveAFB5-activated up-regulation DEGs after 5-day picloram treatment.

Supplementary Table S7. GO enrichment analysis of FveAFB5-repressed down-regulation DEGs after 5-day picloram treatment.
